# Step by step procedures: Degradation of polycyclic aromatic hydrocarbons in potable water using photo-Fenton oxidation process

**DOI:** 10.1016/j.mex.2019.07.011

**Published:** 2019-07-20

**Authors:** Teh Sabariah Binti Abd Manan, Salmia Beddu, Taimur Khan, Wan Hanna Melini Wan Mohtar, Ariyanti Sarwono, Hisyam Jusoh, Nur Liyana Mohd Kamal, Subarna Sivapalan, Abdulnoor A.J. Ghanim

**Affiliations:** a Civil & Environmental Engineering Department, Universiti Teknologi PETRONAS, 32610 Seri Iskandar, Perak Darul Ridzuan, Malaysia; b Department of Civil Engineering, Universiti Tenaga Nasional, Jalan Ikram-Uniten, 43000 Kajang, Selangor Darul Ehsan, Malaysia; c Department of Civil Engineering, Faculty of Engineering, Najran University, P.O Box 1988, King Abdulaziz Road, Najran, Saudi Arabia; d Civil and Structural Engineering Department, Faculty of Engineering and Built Environment, Universiti Kebangsaan Malaysia, 43600 Bangi, Selangor, Malaysia; e Department of Environmental Engineering, Universitas Pertamina, Kebayoran Lama 12220, Jakarta, Indonesia; f Management & Humanities Department, Universiti Teknologi PETRONAS, 32610 Seri Iskandar, Perak Darul Ridzuan, Malaysia

**Keywords:** Step by step procedures: degradation of polycyclic aromatic hydrocarbons from potable water using via photo-Fenton oxidation process, Polycyclic aromatic hydrocarbons, Photo-Fenton oxidation process, Potable water, Water treatment

## Abstract

Polycyclic aromatic hydrocarbons (PAHs) are carcinogenic compounds, composed of two or more fused benzene rings and abundantly found in mixed-use areas. Mixed-use areas consist of dense population, urbanization, industrial and agricultural activities. River pollution are common in mixed-use areas and 98% of Malaysia's fresh water supply originates from surface water. The biological degradation, adsorption and advanced oxidation process were documented as the available PAHs treatment for water. To date, the application of the photo-Fenton oxidation process has been reported for the treatment of PAHs from contaminated soil (review paper), landfill leachate, municipal solid waste leachate, sanitary landfill leachate, aniline wastewater, ammunition wastewater and saline aqueous solutions. As for potable water, the application of Fenton reagent was aided with photo treatment or electrolysis not focusing on PAHs removal.

• The presented MethodsX was conducted for PAHs degradation analysis in potable water samples using photo-Fenton oxidation process.
• The designed reactor for batch experiment is presented.
• The batch experiment consists of parameters like concentration of 17 USEPA-PAHs in the prepared aqueous solution (fixed variable), reaction time, pH and molarity ratio of hydrogen peroxide (H_2_O_2_): ferrous sulfate (FeSO_4_).

## Graphical abstract

**Figure:**
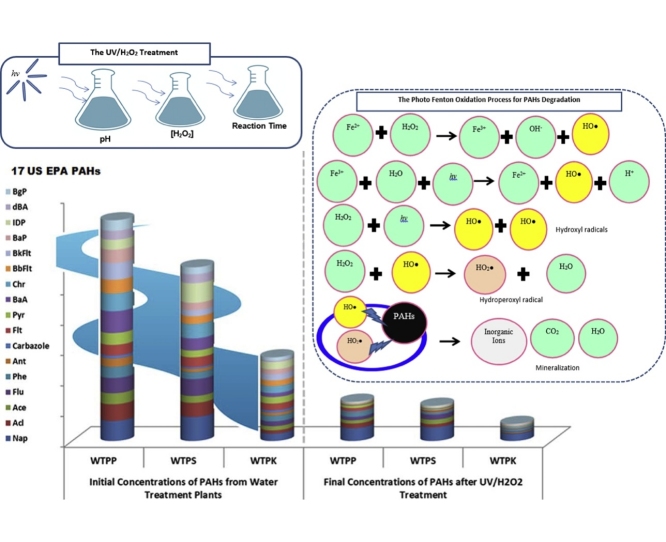

## Specifications table

**Table:** 

Subject area	Engineering
More specific subject area	Environmental Engineering
Method name	Step by step procedures: degradation of polycyclic aromatic hydrocarbons from potable water using via photo-Fenton oxidation process
Name and reference of original method	The preparation method for the aqueous solution containing PAHs was based on the method used by Sakulthaew et al. [1] and Sabaté et al. [2]. The molarity of hydrogen peroxide (H_2_O_2_) and ferrous sulfate (FeSO_4_) was based from Baker et al. [3]. The reaction time, pH and molarity ratio of H_2_O_2_:FeSO_4_ were analyzed for the photo-Fenton oxidation process [4], [5], [6], [7], [8], [9], [10], [11] 1. Baker JR, Milke MW, Mihelcic JR (1999) Relationship between chemical oxygen demand and theoretical oxygen demand for specific classes of organic chemicals. Water Res 33(2): 327–334. 2. Barbusiński K (2009) Fenton reaction-controversy concerning the chemistry. Ecol Chem Eng S 16(3): 347–358. 3. Catalá M, Domínguez-Morueco N, Migens A, Molina R, Martínez F, Valcárcel Y, Mastroianni N, López de Alda M, Barceló D, Segura Y (2015) Elimination of drugs of abuse and their toxicity from natural waters by photo-Fenton treatment. Sci Total Environ 520: 198–205. 4. Ndounla J, Pulgarin C (2014) Evaluation of the efficiency of the photo Fenton disinfection of natural drinking water source during the rainy season in the Sahelian region. Sci Total Environ 493: 229–238. 5. Neyens E, Baeyens J (2003) A review of classic Fenton's peroxidation as an advanced oxidation technique. J Hazard Mater 98:33–50. https://doi.org/10.1016/S0304-3894(02)00282-0. 6. Plakas KV, Sklari SD, Yiankakis DA, Sideropoulos GT, Zaspalis VT, Karabelas AH (2016) Removal of organic micropollutants from drinking water by a novel electro-Fenton filter: Pilot-scale studies. Water Res 9: 183–194. 7. Rubio-Clemente A, Torres-Palma RA, Peñuela GA (2014) Removal of polycyclic aromatic hydrocarbons in aqueous environment by chemical treatments: A review. Sci Total Environ 478:201–225. 8. Sabaté J, Bayona JM, Solanas AM (2001) Photolysis of PAHs in aqueous phase by UV irradiation. Chemosphere 44:119–124. https://doi.org/10.1016/S0045-6535(00)00208-3. 9. Sakulthaew C, Comfort S, Chokejaroenrat C, Harris C, Li X, (2014) A combined chemical and biological approach to transforming and mineralizing PAHs in runoff water. Chemosphere 117:1–9. https://doi.org/10.1016/j.chemosphere.2014.05.041. 10. Sanches S, Leitão C, Penetra A, Cardoso VV, Ferreira E, Benoliel MJ, Crespo MT, Pereira VJ (2011) Direct photolysis of polycyclic aromatic hydrocarbons in drinking water sources. J Hazard Mater 192:1458–1465. 11. Walling C (1975) Fenton's reagent revisited. Acc Cheml Res 6: 125. 12. Miller JS, Olejnik D (2001) Photolysis of polycyclic aromatic hydrocarbons in water. Water Res 35:233–243.
Resource availability	N/A

## Method details

**Fig. 1:**
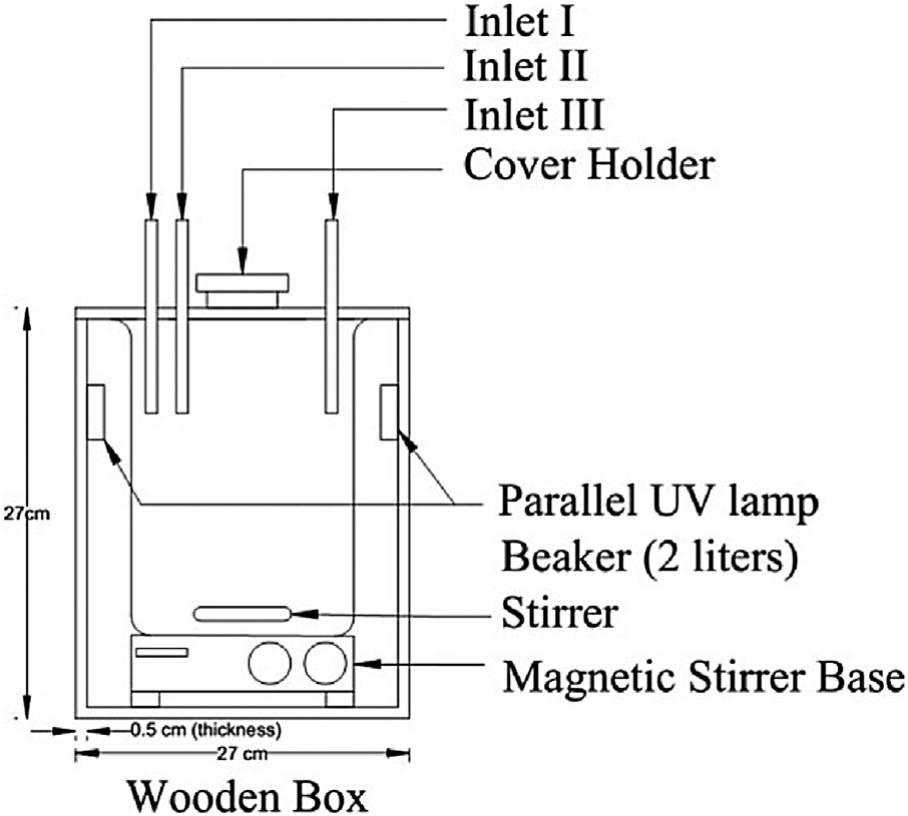
Schematic diagram of the reactor used.

To date, the application of the photo-Fenton oxidation process for PAHs treatment were from contaminated soil (review paper) [12], landfill leachate [13], municipal solid waste leachate [14], sanitary landfill leachate (Tânia et al., 2013), aniline wastewater [15], ammunition wastewater [16] and saline aqueous solutions [17]. As for potable water, the application of Fenton reagent aided with photo treatment [5], [6] or electrolysis [4] were not focusing on PAHs removal. Therefore, this MethodsX is presented to researchers as a step by step procedures of batch experiments for the degradation of PAHs in potable water using photo-fenton oxidation process. The designed reactor for batch experiments is shown in Fig. 1. Design of the reactor in this study was referred to the previous researchers’ designs [18], [19], [20], [21].

Firstly, parameters such as concentration of 17 USEPA-PAHs prepared aqueous solution (4 μg/L of PAHs), the actual pH of the aqueous solution (pH 6.96), molarity of hydrogen peroxide (H_2_O_2_) (0.0075 M) and ferrous sulfate (FeSO_4_) (0.00075 M), room temperature (27 °C), rotational frequency (300 rpm) and UV irradiation (emitting radiation wavelength of 365 nm) should be taken into account. The concentration of 17 USEPA-PAHs was chosen based on the ranges of PAHs concentration documented in Vela et al. [22], Muff and Søgaard [17] and Bertilsson and Widenfalk [23] for their PAHs treatments. The molarity of H_2_O_2_ and FeSO_4_ for the photo-Fenton oxidation process were theoretically calculated and referred to Baker et al. [3]. Table 1 showed the list of parameters and equipments used for the research.

**Table 1:** List of parameters and equipments.

Parameters (Unit)	Equipment
PH	HACH® portable pH meter
Chemical oxygen demand (COD) (0–1500 mg/L))	HACH DR5000 spectrophotometer
Frequency of rotation (300 rpm)	HACH® magnetic stirrer
Temperature (27 °C)	Thermometer
UV irradiation (365 nm)	UV lamp EA-160/FE, 230 V, 0.17 A, Spectronics©
Total organic carbon (TOC) (mg/L)	Total organic carbon analyzer (Shimadzu, Japan)

The analyzed variables were reaction time, pH and molarity ratio (MR) of H_2_O_2_:FeSO_4_. Researchers may conduct the batch experiment starting with observation of reaction time at neutral pH followed by acidic pH and alkaline pH while recording the degradation of parameters under study. The mixing by a magnetic stirrer for complete homogeneity during the reaction is needed. Aliquots must be pipet at the targeted time: (a) 2 mL of aliquot for chemical oxygen demand (COD) and; (b) 20 mL of aliquot for total organic carbon (TOC). If researchers are measuring COD, the pH of the solution need to be adjusted to more than pH 10 to decompose the H_2_O_2_ to oxygen and water to reduce interference in the COD determination. Additional of sodium hydroxide (NaOH) at few drops will be sufficient for the pH adjustment purpose. As photo-Fenton oxidation process involves additional of FeSO_4_, researchers must ensure that the concentration of iron (Fe) should not exceed 1 mg/L in drinking water standards (MOH, 2016) right after every batch experiments.

The degradation of PAHs using photo-Fenton treatment method in potable water samples was studied based on degradations of TOC (along with integrated kinetic rates) and quantification of 17-USEPA PAHs concentrations after the treatment via gas chromatography mass spectrometry (GCMS) analysis.

The determination of TOC was conducted using TOC analyzer (Shimadzu, Japan). The 20 mL volume of collected sample is required for TOC measurement. This measurement is crucial to monitor the degradation of organic compound during the treatment. The use of TOC as proxies for PAHs concentration is justified by Vela et al. [22]. These parameters also were previously reported and used as indications for other water pollutants like 1,2-dichloroethane [24], 2,4-dichlorophenol [19], aniline [15] and ammunition [16] wastewater.

As for the gas chromatography mass spectrometry analysis, the type of PAHs extraction used was solid phase extraction [25], consisted of column HyperSep Retain PEP 60 mg bed weight 3 mL column by Thermo Fisher Scientific Inc (Product Code: 10505905) was used. The cartridge used was C18 Cartridge: 12102052 Bond Elut C18, 500 mg 6 mL, 30 pk from Agilent J & W. Before loading samples, the cartridge was first conditioned with 10 mL DCM/n-hexane (1:3, v/v), then with 10 mL methanol to remove air and leach impurity and then with 10 mL ultrapure water to equilibrate the phase. Next, a 500 mL water sample was loaded at the flow rate of 5.0 mL/min. After loading, the cartridge was kept vacuum for 30 min to remove residual water. The objects retained on the cartridge were eluted by 15 mL/min by 15 mL of DCM/n-hexane (1:3, v/v) at the flow rate of 1 mL/min. The sample re-concentration was done using a type of nitrogen evaporator by Thermo Fisher Scientific namely Reacti-VapTM Evaporators with 9 ports (Product Code: TS-18825). The samples were collected into a test tube and condensed to dryness under gentle flow of nitrogen at room temperature and re-dissolved with 1 mL of DCM:n-hexane (1:3, v/v). Then, the samples were transferred into the 1.5 mL sept vials, ready for GCMS analysis. Samples need to be analyzed within 40 days after the re-concentration ([25]; Agilent Technologies, 2011).

A good calibration for 17 USEPA-PAHS reference standards are required for the quantification of PAHs in the samples before and after treatments. Researchers may test the real water samples of interest before and after treatment too. After calibration, calibration curves must be constructed from scratch by referring on the chromatograms obtained from the calibration (response against ranges of concentrations (eg. 1 μg/L, 2 μg/L and 3 μg/L) for every PAHs tested). For a good validation, the correlation coefficient for calibrations curves (*R*^2^) must be more than 0.95. Researchers may refer on calibration curves of 17 USEPA-PAHs reference standards as per documented in Abd Manan et al. [26].

To sum up, the prescribed method aforementioned above was a recommended steps on the photo-Fenton oxidation process for the degradation of PAHs in potable water conducted by Abd Manan et al. [26]. The optimization products and outcomes were presented by [26]. Authors are hoping that the procedures will be a helpful guide for researchers to conduct water research experiment especially for advanced oxidation processes with some adjustment accordingly wherever necessary.
